# The pre-hospital administration of tranexamic acid to patients with multiple injuries and its effects on rotational thrombelastometry: a prospective observational study in pre-hospital emergency medicine

**DOI:** 10.1186/s13049-016-0314-4

**Published:** 2016-10-10

**Authors:** Nils Kunze-Szikszay, Lennart A. Krack, Pauline Wildenauer, Saskia Wand, Tim Heyne, Karoline Walliser, Christopher Spering, Martin Bauer, Michael Quintel, Markus Roessler

**Affiliations:** 1 Department for Anaesthesiology, University Medical Centre, University of Göttingen, Robert-Koch-Straße 40, 37075 Göttingen, Germany; 2 Department for Trauma Surgery and Orthopaedics, University Medical Centre, University of Göttingen, Robert-Koch-Straße 40, 37075 Göttingen, Germany

**Keywords:** Trauma, Fibrinolysis, Thrombelastometry, Coagulopathy, Tranexamic acid, Pre-hospital care

## Abstract

**Background:**

Hyperfibrinolysis (HF) is a major contributor to coagulopathy and mortality in trauma patients. This study investigated (i) the rate of HF during the pre-hospital management of patients with multiple injuries and (ii) the effects of pre-hospital tranexamic acid (TxA) administration on the coagulation system.

**Methods:**

From 27 trauma patients with pre-hospital an estimated injury severity score (ISS) ≥16 points blood was obtained at the scene and on admission to the emergency department (ED). All patients received 1 g of TxA after the first blood sample was taken. Rotational thrombelastometry (ROTEM) was performed for both blood samples, and the results were compared. HF was defined as a maximum lysis (ML) >15 % in EXTEM.

**Results:**

The median (min-max) ISS was 17 points (4–50 points). Four patients (15 %) had HF diagnosed via ROTEM at the scene, and 2 patients (7.5 %) had HF diagnosed via ROTEM on admission to the ED. The median ML before TxA administration was 11 % (3–99 %) vs. 10 % after TxA administration (4–18 %; *p* > 0.05). TxA was administered 37 min (10–85 min) before ED arrival. The ROTEM results before and after TxA administration did not significantly differ. No adverse drug reactions were observed after TxA administration.

**Discussion:**

HF can be present in severely injured patients during pre-hospital care. Antifibrinolytic therapy administered at the scene is a significant time saver. Even in milder trauma fibrinogen can be decreased to critically low levels. Early administration of TxA cannot reverse or entirely stop this decrease.

**Conclusions:**

The pre-hospital use of TxA should be considered for severely injured patients to prevent the worsening of trauma-induced coagulopathy and unnecessarily high fibrinogen consumption.

**Trial registration:**

ClinicalTrials.gov ID NCT01938768 (Registered 5 September 2013).

## Background

Major trauma remains a leading cause of death worldwide, particularly in younger people, with haemorrhage accounting for approximately one third of all trauma-related deaths [1, 2]. Trauma-associated coagulopathy occurs early and is an important contributor to mortality in severely injured patients [3]. The loss of coagulation factors, together with the extensive activation of the coagulation system due to release of tissue factor from injured body regions can cause life-threatening coagulopathy, which is an independent risk factor for trauma-associated mortality [4, 5]. Hypothermia, acidosis, dilution after fluid resuscitation and other factors contribute to trauma-associated coagulopathy [5].

The activation of coagulation leads to the stimulation of fibrinolysis by transformation of plasminogen to plasmin [6]. As a result of the massive coagulation activation after major trauma, the physiological process of fibrinolysis can exceed fibrin formation, which can induce the breakdown of freshly formed clots. This phenomenon, known as hyperfibrinolysis (HF), impedes the formation of functional clots and can become a major contributor to the consumption of coagulation factors, in particular fibrinogen. Fibrinogen is the coagulation factor that first reaches critically low levels in bleeding trauma patients [7]. Therefore, HF contributes to the vicious circle of haemorrhage, fibrinolytic activation and fibrinogen consumption in bleeding trauma patients. However, the exact point at which fibrinolysis extents fibrin formation and HF begins is unknown.

In a recent study of fibrinolysis, HF was diagnosed via thrombelastometry (TEM) in 5 % of all trauma patients; however 57 % showed moderate fibrinolysis with elevated plasmin-antiplasmin (PAP) complex levels [8]. Other authors have reported that between 6 and 16 % of trauma patients present with severe HF visible in TEM [9–11]. HF in TEM is defined as a maximum lysis (ML) exceeding 15 % of the maximum clot firmness (MCF) [10]. The incidence and extent of HF in TEM and measured via PAP correlates with injury severity. HF is also an independent contributor to mortality in major trauma patients [8, 10, 12].

Tranexamic acid (TxA) inhibits fibrinolysis by blocking the binding of lysine to plasminogen [6]. TxA was first introduced as an antifibrinolytic drug in cardiovascular and orthopaedic surgery. The results of the large CRASH-2 trial showed that the administration of TxA within the first three hours after hospital admission reduced mortality in trauma patients [2, 13]. Mortality rates were lowest among patients who received TxA within the first hour after hospital admission, and authors concluded that TxA should be given as early as possible to bleeding trauma patients [2]. Consequently, the early administration of TxA must be included in the initial clinical management of trauma patients [14]. Based on the assumption that TxA should be administered as early as possible to bleeding trauma patients, TxA has been implemented in civilian and military emergency medical services (EMSs) for immediate on-the-scene administration [15, 16]. Nevertheless, a recent review by Schoechl et al. revealed significant gaps in knowledge regarding the effects of pre-hospital TxA administration. Apart from data obtained in military emergency medicine, no current evidence supports the use of TxA in pre-hospital emergency medicine [17].

TxA was implemented in the EMS of Göttingen, Germany in December 2013. We conducted a prospective observational study to investigate the effects of early pre-hospital TxA administration among patients with multiple injuries. Blood samples from trauma patients were collected before the pre-hospital administration of TxA and after arrival at the emergency department (ED), and the results of the TEM measurements from both samples were compared. We aimed to answer the following questions: (i) Is HF detectable by TEM at such an early stage after major trauma? (ii) What are the effects of pre-hospital TxA administration on the coagulation system?

## Methods

### Study design and data collection

This prospective observational study was part of a research project investigating the effects of early TxA administration during pre-hospital care that was approved by the Ethics Committee of the Medical Faculty of the University of Göttingen (approval number 16/4/13). Informed consent was obtained from patients following their recovery from acute trauma. In cases of longer lasting unconsciousness or disorientation, informed consent was obtained from an officially appointed guardian following the suspected will of the patient. Based on the approval of the Ethics Committee, we did not exclude patients who died during the first 72 h after trauma. This study was registered at www.clintrials.gov (NCT01938768).

Between December 2013 and August 2015, adult trauma patients who were pre-hospitally attended by an EMS emergency physician (EP) of the Department for Anaesthesiology of the University Medical Centre of Göttingen were included in this study. The inclusion criteria were a diagnosis of major trauma with a pre-hospitally assumed injury severity score (ISS) ≥16 points [18] and the pre-hospital administration of 1 g TxA. The exclusion criteria were age <16, refusal to consent, TEM measurement delayed >4 h, TEM measurement <50 min, and missing/insufficient blood samples. Patients who died before hospital arrival were considered as intention-to-treat and therefore not excluded.

TxA was implemented at the Göttingen EMS in December 2013, and the administration of 1 g of TxA is part of the routine management of trauma patients at risk for bleeding. For the rotational TEM (ROTEM) measurements, a 3-ml blood sample was taken from each patient who met the inclusion criteria as soon as intravenous access was established and before the 1 g of TxA was administered. A second 3-ml blood sample was taken immediately after arrival at the ED, together with the blood samples taken for routine laboratory investigations. Sample tubes containing 0.3 ml of buffered 3.2 % trisodium citrate (S-Monovette®, Sarstedt AG & Co, Nümbrecht, Germany) were used for blood sampling.

The data regarding patient demographics, time of injury, time of EMS attendance, time of TxA administration, time of hospital arrival and the mechanisms of injury were collected prospectively.

### ROTEM analyses

The ROTEM measurements of both blood samples were started within 2 h after arrival at the hospital using a ROTEM delta® analyser (Tem International GmbH, Munich, Germany). The working principle and parameters of ROTEM were described previously [19]. From each blood sample, four separate ROTEM assays were started: EXTEM (coagulation activation by tissue factor from rabbit brain), INTEM (coagulation activation by ellagic acid), FIBTEM (coagulation activation by tissue factor and inhibition of platelet function by cytochalasin D) and APTEM (coagulation activation by tissue factor and inhibition of fibrinolysis by TxA). The liquid reagents star-tem®, ex-tem®, in-tem®, fib-tem® and t ap-tem® (Tem International GmbH, Munich, Germany) were used for all ROTEM analyses. Blood and reagents were pipetted using the electronic pipette program of the ROTEM device. Blood samples were stored in the 37 °C warming plate of the ROTEM device until analysis.

The following parameters were measured and analysed: clotting time (CT, sec), MCF (mm), and ML (%). The maximum clot elasticity (MCE, no dimension) was calculated as MCE = (MCF – 100)/(100 – MCF). HF in ROTEM was defined as an ML in EXTEM of >15 %.

### Laboratory investigations and blood gas analyses

The following laboratory parameters were analysed from the blood samples taken shortly after hospital arrival: prothrombin time index (PTI, %), activated partial thromboplastin time (aPTT, sec), fibrinogen concentration (measured using the Clauss method, optical read-out, g/L), haemoglobin (Hb, g/dL), platelet count (PC, 10^3^/μL), calcium (Ca^2+^, mmol/L), and PAP complex (μg/L). Blood samples for standard laboratory investigations were taken as part of the clinical routine and analysed at the central laboratory facilities of the University Medical Centre of Göttingen. The following assays were used for the laboratory investigation: PTI (RecombiPlasTin 2G, Instrumentation Laboratory Company, Bedford, MA, USA), aPTT (SynthASil, Instrumentation Laboratory Company, Bedford, MA, USA), fibrinogen (Q.F.A. Thrombin [Bovine], Instrumentation Laboratory Company, Bedford, MA, USA), Hb and PC (CELL-DYN Sapphire, Abbott Laboratories, Abbott Park IL, USA), Ca^2+^ (AEROSET Calcium assay, Abbot Laboratories, Abbott Park, IL, USA), PAP (PAP micro ELISA, DRG Instruments GmbH, Marburg, Germany). A blood gas analysis (BGA) is part of the initial laboratory investigation of trauma patients upon arrival to the ED. pH and lactate values were obtained from the initial BGA (GEM 4000 blood gas analyser, Instrumentation Laboratory Co., Bedford, MA, USA).

### Statistical analyses

Descriptive analyses and significance testing were performed using Statistica 10 (StatSoft Inc., Tulsa, USA). All parameters were investigated for normal distribution using the Kolmogorov-Smirnov test of normality. Significance testing was performed using the independent-samples *t*-test for normally distributed parameters and the Mann-Whitney *U* test was applied for non-normally distributed parameters. *P*-values < 0.05 were considered as significant.

## Results

Thirty-two patients were eligible for this study. Five patients were excluded (one withdrew consent and four had underfilled or diluted blood samples). The remaining 27 patients were enrolled, and their data were analysed according to the study protocol. Table 1 summarises the characteristics of the study population and the laboratory findings upon hospital arrival. No adverse drug reactions were observed during and after administration of TxA. No major thromboembolic events were observed within 30 days after hospital admission.

**Table 1:** Patient demographics, injury severity, mechanisms of injury and laboratory results

	Patients (*n* = 27)
Age, median [min-max], years	50 [16–88]
Female, *n* (*%*)	5 (18.5)
Death before ICU admission, *n* (%)	1 (3.5)
30 d-survival, *n* (*%*)	24 (89)
ISS^a^, median [min-max], points	17 [4–50]
ISS ≥16 points, *yes*, *n* (*%*)	15 (55.5)
NISS^a^, median [min-max], points	17 [8–57]
NISS ≥16 points, *yes*, *n* (*%*)	18 (66.7)
Mechanisms of injury, *n* (*%*)	
Road traffic accident (RTA)	14 (52)
RTA – motorcyclist	5 (18.5)
Fall	7 (26)
Other	1 (3.5)
First blood gas analysis on ED arrival, median [min-max]	
pH	7.32 [6.84–7.39]
Haemoglobin, g/dL	12.9 [4.3–18.1]
Lactate, mmol/L	1.6 [1.1–15.6]
Base excess, mmol/L	−0.3 [−18.9–4.8]
Calcium, mmol/L	1.2 [0.98–1.59]
Laboratory analyses on ED arrival, median [min-max]	
PTI, %	91 [5–132]
aPTT, seconds	26 [21–180]
Fibrinogen, g/L	1.89 [0.47–4.79]
Platelets, ×10^3^/μL	199 [15–354]
PAP complex^b^, μg/L	1837 [508–4815]

^a^ISS according to the final results of clinical investigation and x-rays (incl. CT scans)

^b^Missing data in three patients

The median time interval from injury to the arrival of the TxA-carrying EP was 18 min (min-max: 4–65 min), and TxA was administered a median time of 15 min (7–53 min) later. Patients reached the ED 56 min (25–128 min) after the arrival of the EP on the scene. In median TxA was given 37 min (10–85 min) prior to hospital arrival. Table 2 summarises these results.

**Table 2:** Time flow of EMS response, TxA administration and ED arrival

	Median time [min, max]
Time to EP arrival (after injury)	18 min [4, 65]
Time to TxA administration (after injury)	32 min [17, 85]
Time to TxA administration (after EP arrival)	15 min [7, 53]
Time to hospital arrival (after injury)	74 min [31, 140]
Time to hospital arrival (after EP arrival)	56 min [25, 128]
Time to hospital arrival (after TxA administration)	37 min [10, 85]

**Fig. 1:**
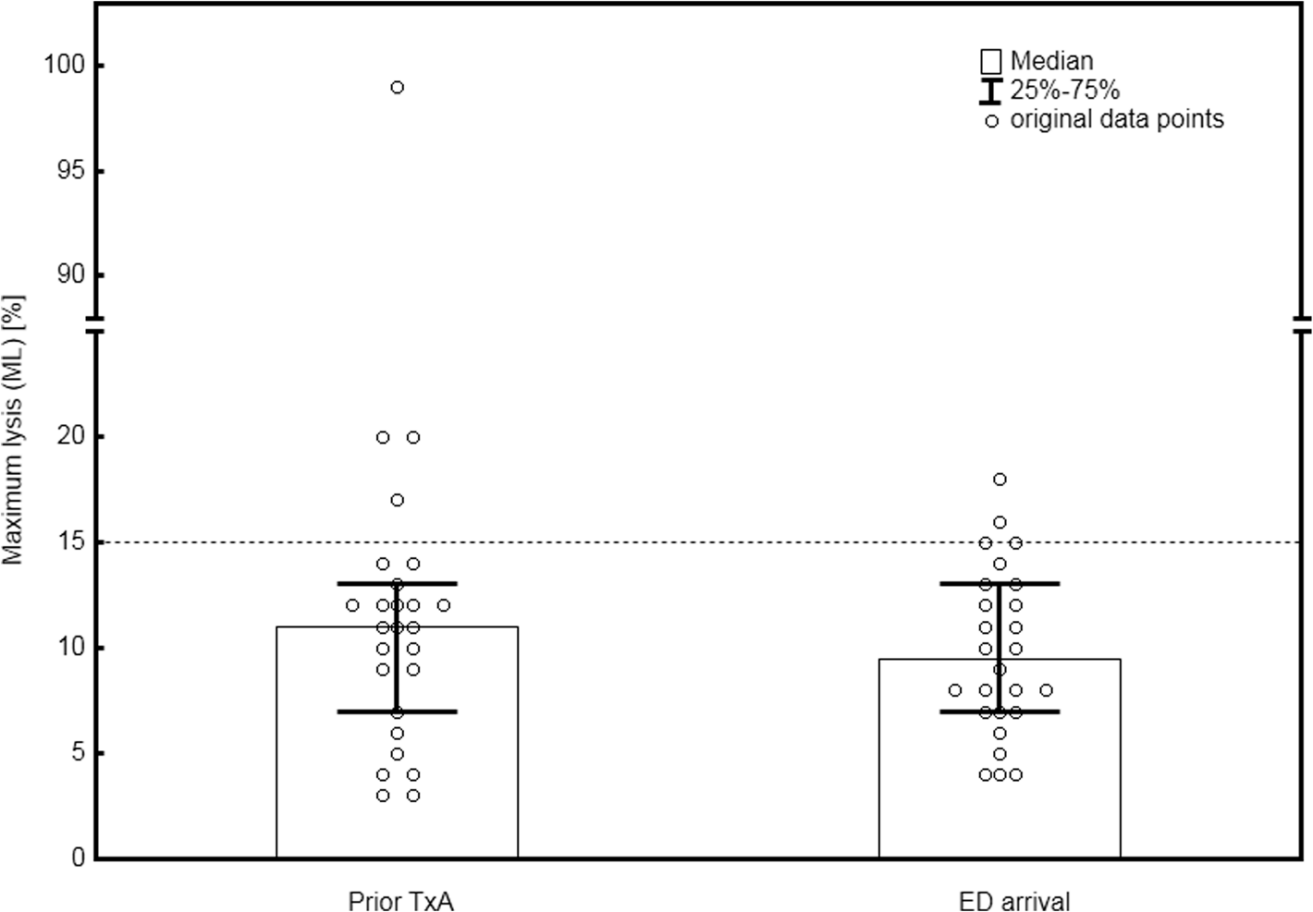
ML in ROTEM on the scene (prior to TxA administration) and after ED arrival. No significant differences were found before and after the administration of TxA (*p* > 0.05). In one patient no clot formatted in neither blood sample and therefore ML was not available

**Fig. 2:**
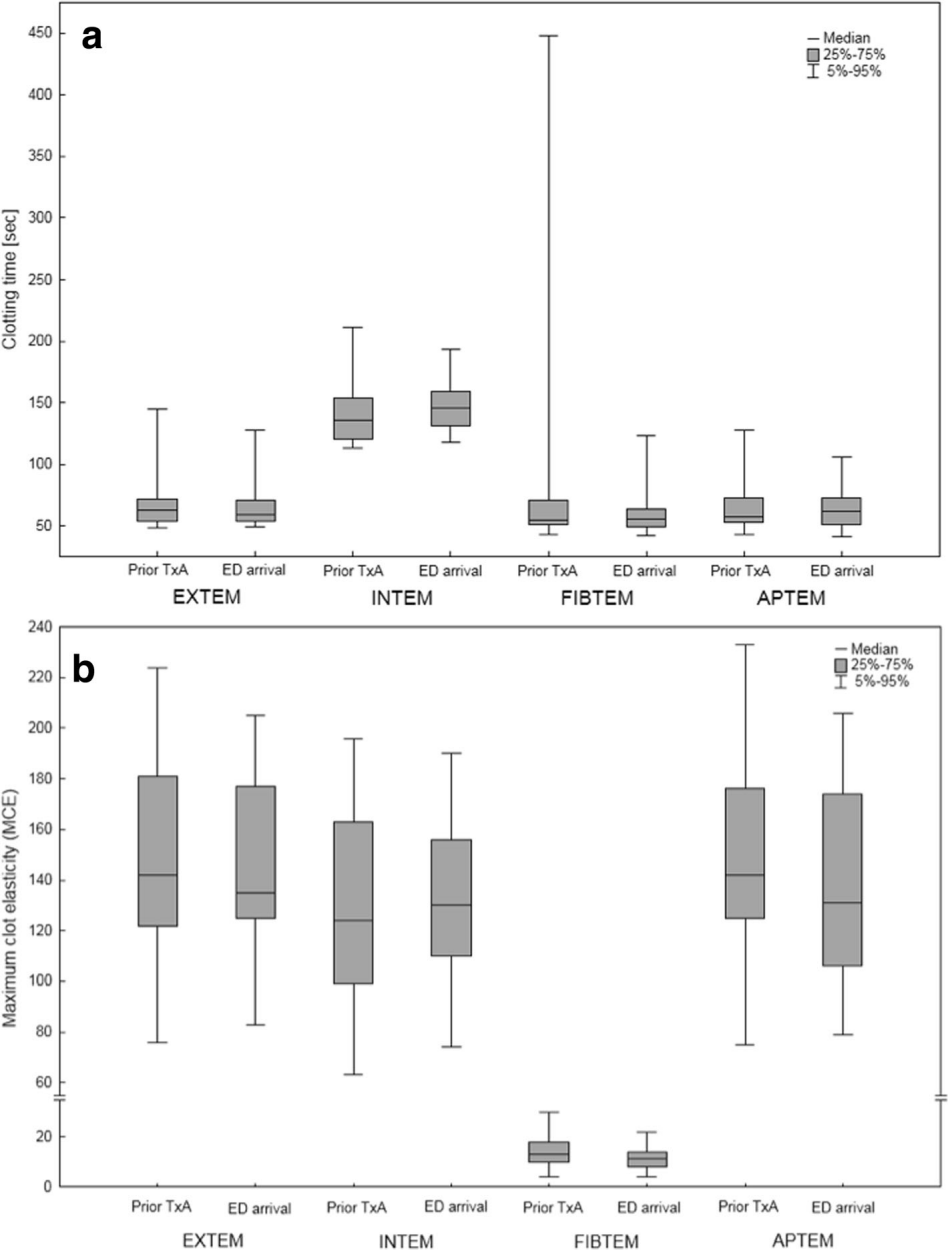
Results of the ROTEM analyses on the scene (prior to TxA administration) and after ED arrival: **a** CT in EXTEM, INTEM, FIBTEM and APTEM **b** MCE in EXTEM, INTEM, FIBTEM and APTEM. No significant differences were found before and after the administration of TxA (*p* > 0.05)

The median duration of the ROTEM measurements was 90:14 min:sec (52:25–90:29 min:sec). Prior to the administration of TxA, HF in EXTEM was found in four patients (15 %). The median ML in EXTEM prior to receiving TxA was 11 % (3–99 %) (see Fig. 1). Upon hospital arrival, HF in EXTEM was visible in two patients (7.5 %), and the median ML was 10 % (4–18 %). The differences in ML before and after the pre-hospital administration of TxA were not significant (*p* > 0.05). Figure 2 summarizes these results. Table 3 summarizes the results of the TEM analyses.

**Table 3:** Results of the ROTEM analyses

	Prior TxA	ED arrival	*p*-value
EXTEM			
CT, median [min-max], sec	63 [47–348]	59 [44–2659]	0.97
MCF, median [min-max], mm	59 [16–72]	57 [0–68]^a^	0.72
MCE, median [min-max]	143 [19–251]	136 [0–214]^a^	0.71
ML median [min-max], %	11 [0–99]	10 [4–18]	0.68
INTEM			
CT, median [min-max], sec	136 [106–1039]	146 [107–2848]	0.22
MCF, median [min-max], mm	55 [10–68]	56 [0–68]^a^	0.75
MCE, median [min-max]	124 [11–128]	130 [0–211]^a^	0.89
ML median [min-max], %	11 [2–81]	9 [3–18]	0.63
FIBTEM			
CT, median [min-max], sec	55 [38–3600]^a^	56 [42–3600]^a^	0.94
MCF, median [min-max], mm	12 [0–23]^a^	10 [0–21]^a^	0.29
MCE, median [min-max]	13 [0–31]^a^	11 [0–27]^a^	0.26
ML median [min-max], %	1 [0–97]	0 [0–10]	0.10
APTEM			
CT, median [min-max], sec	58 [42–418]	62 [40–1349]	0.78
MCF, median [min-max], mm	59 [15–71]	57 [5–67]	0.56
MCE, median [min-max]	142 [17–251]	133 [5–207]	0.54
ML, median [min-max], %	11 [0–20]	11 [3–19]	0.80

^a^if no clot formation was visible in TEM analyses CT was defined as 3600 sec (minimal duration of TEM analyses). MCF and MCE were defined as 0

With a ML in EXTEM of 99 % prior to TxA administration one patient showed complete clot breakdown in ROTEM. This patient had the most severe HF in our study and the effects of the early TxA administration are therefore described more in detail: A TxA carrying EP attended this patient ten minutes after the traumatic incident. 15 min later 1 g of TxA was administered (the blood sample for the first ROTEM had been collected before). The ED was reached 39 min later (64 min after injury). The patient had an ISS of 45. In the second ROTEM analysis (after 1 g of TxA) the ML in EXTEM was 4 %. The MCE in FIBTEM in the initial ROTEM was 8 and 7 after hospital arrival.

## Discussion

The present study investigated (i) whether severe HF occurs during the initial on-the-scene management of trauma patients and (ii) the effects of a pre-hospital TxA administration on the coagulation system. As such, we compared the TEM analyses of the blood samples from trauma patients taken before the pre-hospital administration of TxA and after arrival at the ED.

Overall, we found that HF was present in 15 % of patients prior to the administration of TxA. In our study, three of four patients with ML >15 % had an ISS of ≥17 points. One patient with an ISS of eight points had an ML of 20 % before the administration of TxA. Several authors reported of associations between ISS and the incidence and degree of HF [8, 10, 11]. Our findings support these observations, even during the early stages following injury. Fibrinolytic activation seems to be part of the early changes during trauma-associated coagulopathy and therefore contributes to fibrinogen consumption from the beginning of the disorder. In their recently published study, Theusinger et al. reported that 10 % of trauma patients have a ML >15 % at the scene [11]. Our data corroborate their observation that severely injured patients can present with significant HF only minutes after injury. Although only 55 % of the patients in the study had an ISS ≥16 points we did observe critically low fibrinogen levels in the first ROTEM analyses. Without treatment, the extent of fibrinolysis and fibrinogen consumption will increase during pre-hospital treatment and patient transportation, which was also demonstrated by Theusinger et al., who did not administer TxA prior to hospital admission, resulting in an HF rate that was 6 % higher upon hospital arrival [11]. In contrast, the rate of HF was halved in our study, and only mild forms of HF with a maximum ML of 18 % were present upon hospital arrival. Because of the ongoing blood loss in many situations the pre-hospital administration of TxA will not be able to stop or even reverse the decrease of fibrinogen in bleeding trauma patients. It can, however, help to prevent unnecessary fibrinogen consumption caused by fibrinolysis.

In our study, 1 g of TxA was given to patients with multiple injuries immediately after intravenous access was established and the study blood samples had been taken. Although TxA has no current marketing indication for use among major trauma patients, it is commonly used in clinical practice, and its administration is recommended by current guidelines [20]. The recently published NICE guidelines on the initial assessment and management of major trauma patients do recommend that TxA should be given as soon as possible. Moreover, based on the findings of the CRASH-2 trial, TxA administration more than three hours after *injury* (not hospital admission) is discouraged among major trauma patients [2, 20]. Patients in our study received TxA in median 32 min after injury. The median time until hospital arrival was 74 min after injury. To our knowledge, no data are available regarding the timing of TxA administration in pre-hospital emergency medicine. Our results showed that the on-scene administration of TxA leads to a significant time advantage for trauma patients and supports the use of TxA as soon as possible after injury. In some trauma patients (e.g., entrapped patients after road traffic accidents and those requiring long transportation distances), early administration of TxA might in fact be the only option to ensure administration within three hours after injury as recommended by the guidelines.

Antifibrinolytic therapy should be given as soon as possible when trauma patients are at risk of haemorrhage and coagulopathy. The fast and secure identification of such patients in pre-hospital care is therefore of particular importance. TEM or fibrinolysis-specific laboratory tests are not available in pre-hospital care. Thus, alternative markers are needed that reliably identify high-risk trauma patients.

Injury severity scoring systems such as the ISS and NISS can be used as surrogates. Both can be estimated during the pre-hospital management of trauma patients, but due to the unavailability of radiological investigations, however, the risk for over- and underestimation is high. Our study included trauma patients who were initially diagnosed with an ISS ≥16. According to our actual routine management, these patients were considered as at risk for haemorrhage and trauma-induced coagulopathy and therefore received 1 g of TxA during pre-hospital treatment. Based on the results of the clinical and radiological investigations that were performed after hospital admission, this diagnosis was correct for 55.5 % of our patients. Obviously, the ISS was significantly overestimated on the scene. Several authors have reported that both EPs and paramedics overestimate injury severity in pre-hospital care. Overestimation rates of up to 90 % have been reported in the literature [21]. The overestimation rate of 44.5 % regarding predicted injury severity in our study was slightly better than those in the literature. More research is needed to determine which trauma patients benefit most from pre-hospital antifibrinolytic therapy. Our data suggest that the estimation of injury severity by an EP during pre-hospital management is poorly correlated with the actual ISS and therefore does not appear to be a suitable parameter for identifying high-risk patients. Haemodynamic instability in trauma patients requiring vasopressors, epinephrine, or both might be considered as a trigger for the pre-hospital use of TxA because high concentrations of epinephrine and vasopressin are known to cause significant release of tissue plasminogen activator (t-PA) from endothelial cells [22].

The results of the TEM analyses before and after TxA administration did not show any significant differences with regard to any of the relevant TEM parameters. However, we were able to show that ML improved in all patients with severe HF on the scene until hospital arrival. Theusinger et al. reported significant changes in the coagulation parameters between the blood samples that were taken at the scene and those that were taken at hospital admission. They investigated both plasma-based coagulation parameters and ROTEM in trauma patients. In their study, all coagulation parameters significantly deteriorated over time (at the scene vs. hospital arrival) [11]. In our study, no significant changes were identified between the investigated TEM parameters, although the median time to hospital arrival for our patients was 29 min later than that in Theusinger et al. The median ISS of 17 points was the same in both studies. However, because no coagulation-specific or antifibrinolytic therapy was administered in their study, the authors suggested that a “significant consumption of fibrinogen” (and other coagulation factors) and “progressive fibrinolysis” took place between both measurements [11]. In our study, we did not observe any significant deterioration with regard to the relevant TEM parameters following the administration of 1 g of TxA. In particular, no significant decreases in MCE were observed in EXTEM, INTEM and FIBTEM, nor did the CTs increase in EXTEM and INTEM. Despite the limitations of our small sample size and the heterogeneity of our study population, our results suggest that early antifibrinolytic therapy leads to less fibrinolytic activation, and therefore lower fibrinogen consumption, resulting in an improved function of the coagulation system at hospital admission.

Our study has several limitations. First, our sample size was small. Thus, no subgroup analyses could be performed (e.g., by different classes of injury severity). Second, the administration of TxA in our study was part of the routine management of trauma patients and therefore random assignment and investigator blinding were not possible. One team member took the blood samples at the scene after arrival of the TxA-carrying EP and during pre-hospital treatment. Although no information was obtained regarding the fluid and pharmacological therapy until the first blood sample was taken, no patient received antifibrinolytic therapy before the arrival of the TxA-carrying EP. Furthermore, other than the administration of TxA, no information was obtained regarding the fluid and pharmacological therapy during pre-hospital treatment. TxA is the only coagulation-specific medication carried by the Göttingen EMS. No other blood products or coagulation factors were administered during pre-hospital care. We detected severe HF by assessing the ML in the TEM analyses. As Raza et al. described, the PAP complex is a more sensitive marker for mild and moderate HF [8]. In our study, PAP was measured only at hospital arrival. In order to perform this study without disturbing patient treatment, we decided that no more than one blood sample container should be taken during the initial treatment. Therefore, we were unable to measure PAP before and after the administration of TxA because this would have required another separate blood sample.

To our knowledge, this study is the first that prospectively investigated the influence of early pre-hospital antifibrinolytic therapy on trauma patients in a non-military environment. Our results found a significant time advantage associated with the pre-hospital administration of TxA and suggest that the resulting lower fibrinolytic activation at the onset of the development of trauma-associated coagulopathy leads to a better functioning of the coagulation system upon hospital arrival. More research is needed to investigate the influence of pre-hospital antifibrinolytic therapy on other outcome parameters, such as mortality, morbidity, therapy costs or the need for blood products.

## Conclusions

Severe HF can be present in trauma patients only minutes after injury. Even in milder trauma (ISS <16 points) fibrinogen can be decreased to critically low levels. Early administration of TxA cannot reverse or entirely stop this decrease. However, the pre-hospital administration of TxA leads to a significant time advantage in the initiation of antifibrinolytic therapy and probable is a highly effective therapy of HF before hospital arrival. Additional research is required to assess the effect of pre-hospital antifibrinolytic therapy on the outcome parameters. However, the use of TxA during pre-hospital care should be considered for severely injured patients to break the vicious circle of haemorrhage, fibrinolytic activation and fibrinogen consumption as early as possible.

## Abbreviations

aPTT: Activated partial thromboplastin time
BGA: Blood gas analysis
CT: Clotting time
ED: Emergency department
EMS: Emergency medical service
EP: Emergency physician
Hb: Haemoglobin
HF: Hyperfibrinolysis
ISS: Injury severity score
MCE: Maximum clot elasticity
MCF: Maximum clot firmness
ML: Maximum lysis
NICE: National Institute for Health and Clinical Excellence of the United Kingdom
PAP: Plasmin-antiplasmin
PC: Platelet count
PTI: Prothrombin time index
ROTEM: Rotational thrombelastometry
TEM: Thrombelastometry
TxA: Tranexamic acid
